# Supplementary material for: A quantitative approach for the analysis of clinician recognition of acute respiratory distress syndrome using electronic health record data
Source: PLoS One. 2019 Sep 20;14(9):e0222826. doi: 10.1371/journal.pone.0222826 (PMC6754155; doi:10.1371/journal.pone.0222826)
Supplement: S1 Methods — (DOCX) [file pone.0222826.s001.docx]

**SUPPLEMENTAL METHODS**

**Multivariable model selection – ARDS cohort**

Seven models were constructed using all combinations of PBW, Documentation, and lowest P/F ratio as independent variables and a P/F ratio Documentation interaction term (S3 Table). In each model, the continuous variables were scaled between 0 and 1. AIC and BIC were calculated for each model to select the “best” model. AIC and BIC were calculated using the python package *statsmodels* (version 0.6.1).
