# Supplementary figures and images for: A quantitative approach for the analysis of clinician recognition of acute respiratory distress syndrome using electronic health record data
Source: PLoS One. 2019 Sep 20;14(9):e0222826. doi: 10.1371/journal.pone.0222826 (PMC6754155; doi:10.1371/journal.pone.0222826)

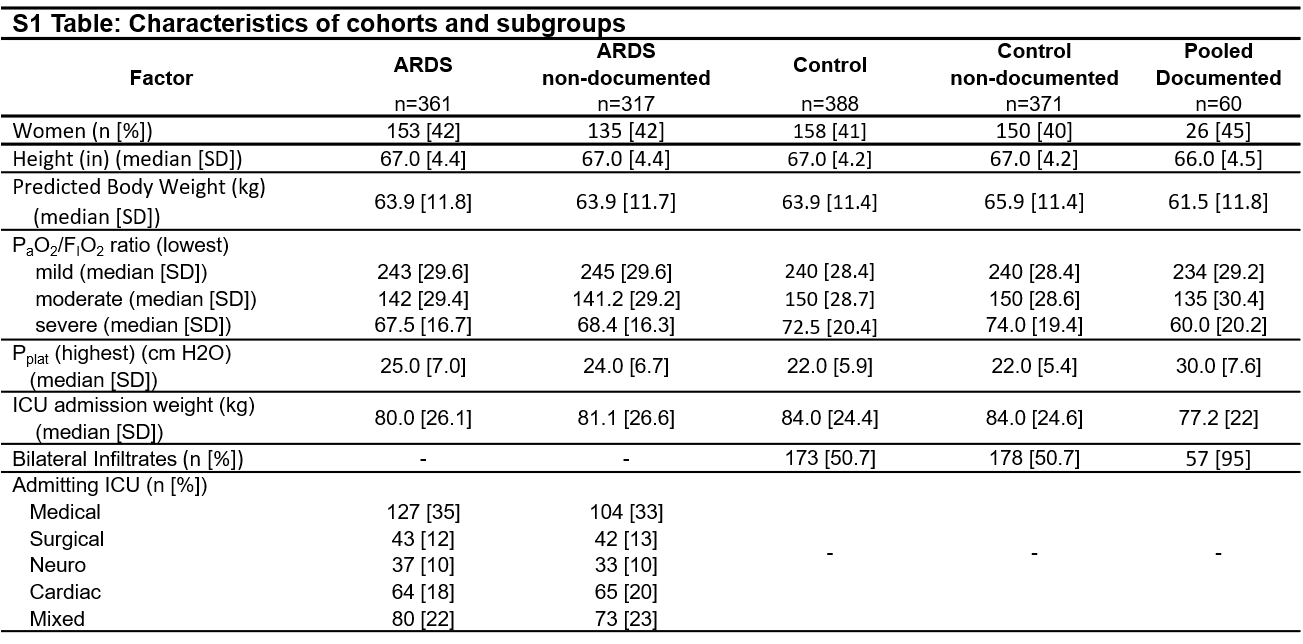

Supplement: S1 Table — (TIF) [file pone.0222826.s002.tif]

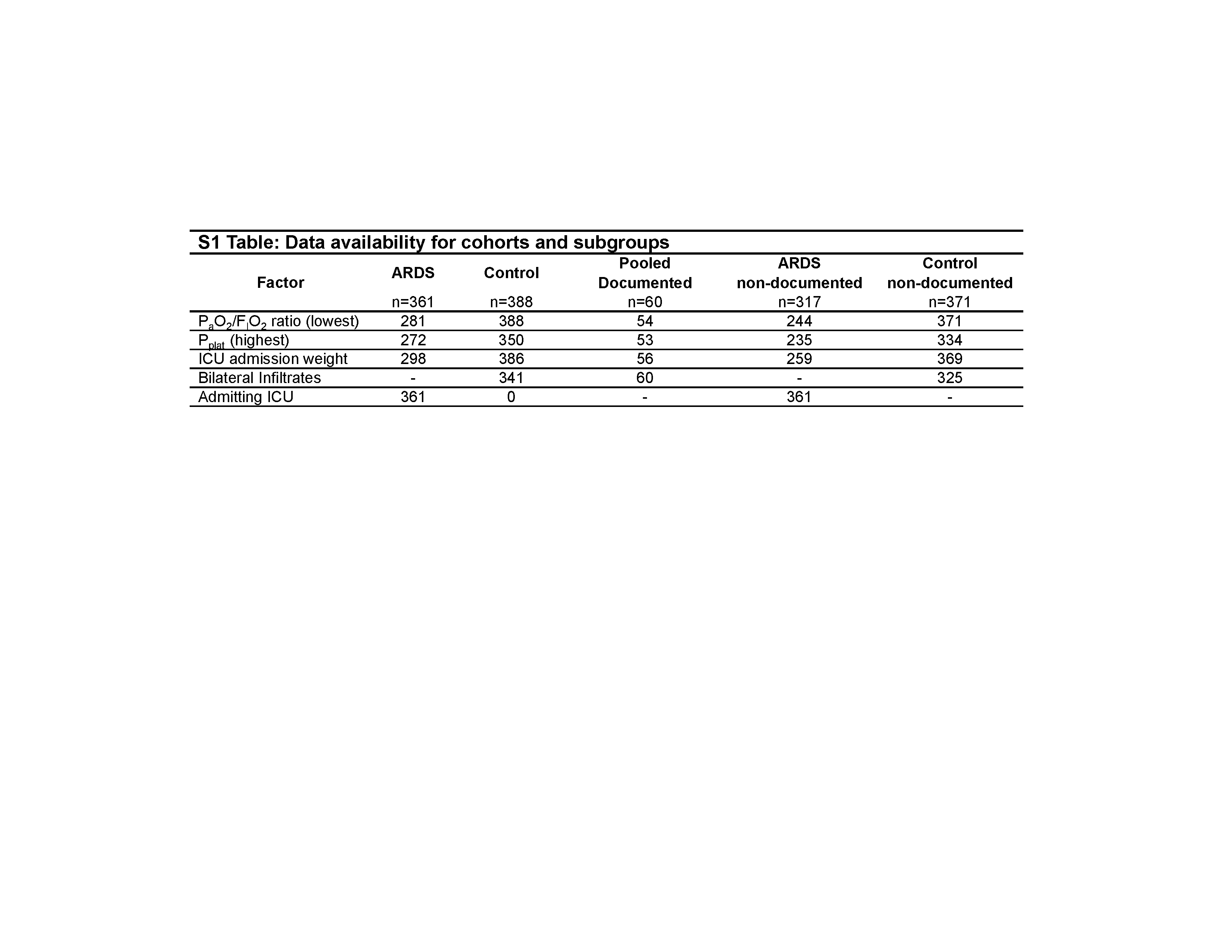

Supplement: S2 Table — (TIF) [file pone.0222826.s003.tif]

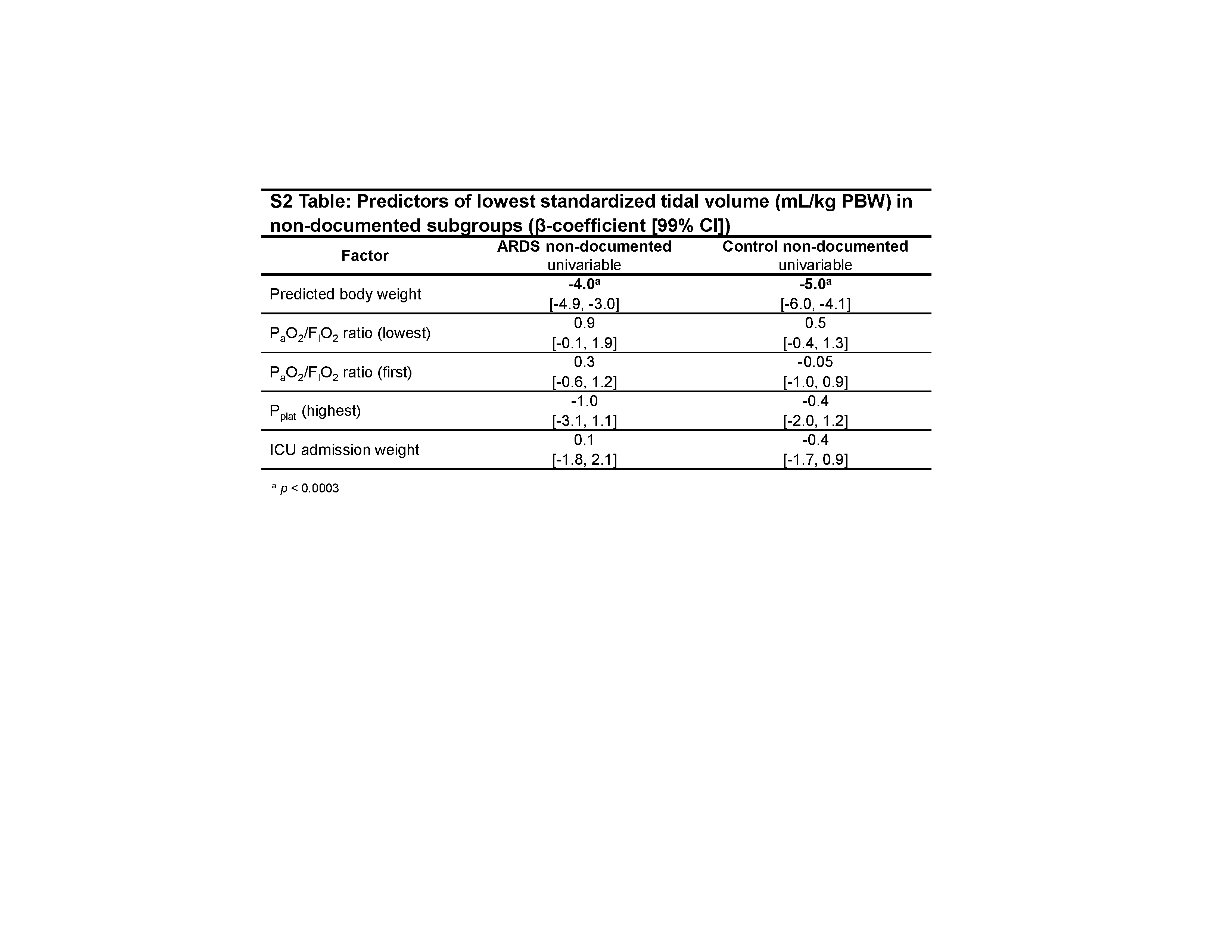

Supplement: S3 Table — (TIF) [file pone.0222826.s004.tif]

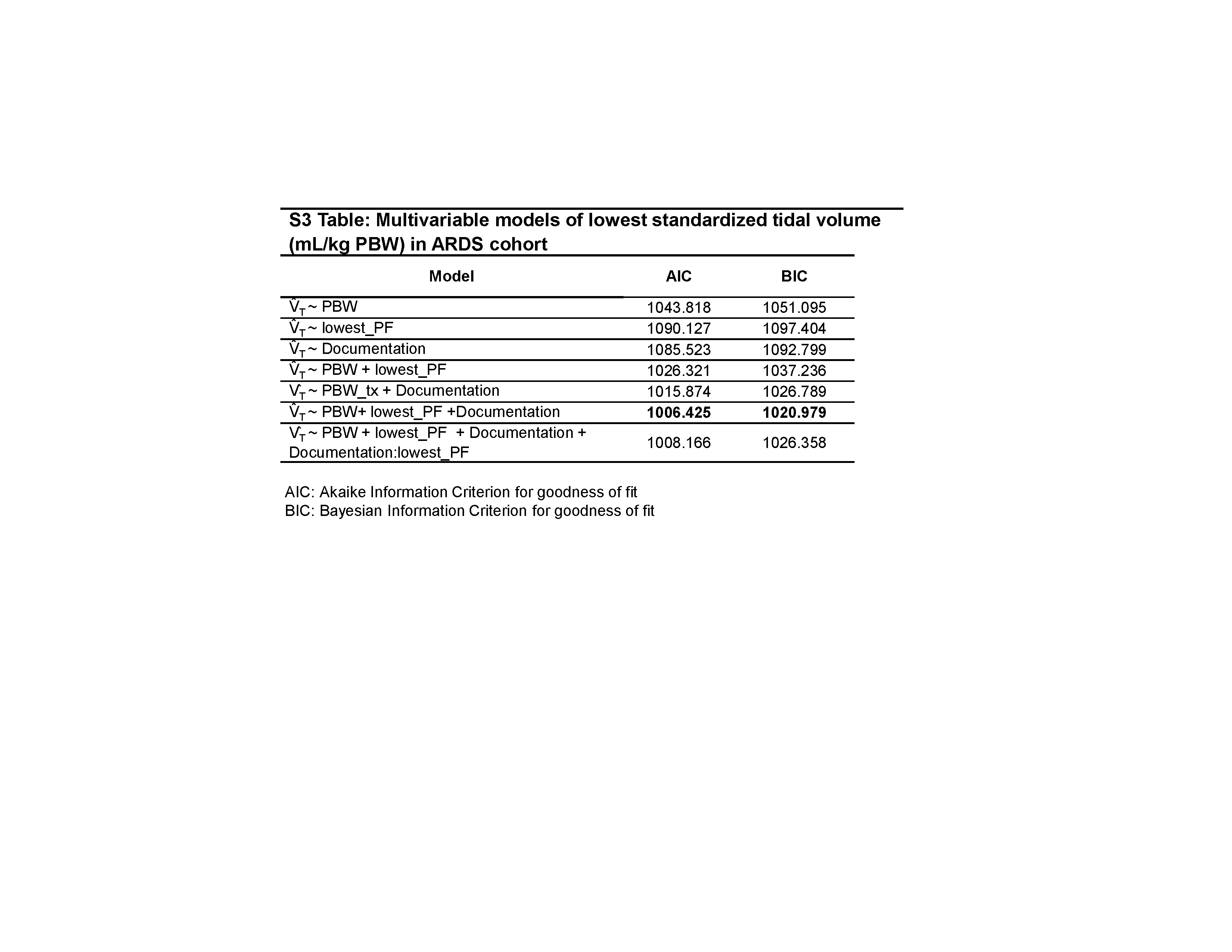

Supplement: S4 Table — (TIF) [file pone.0222826.s005.tif]

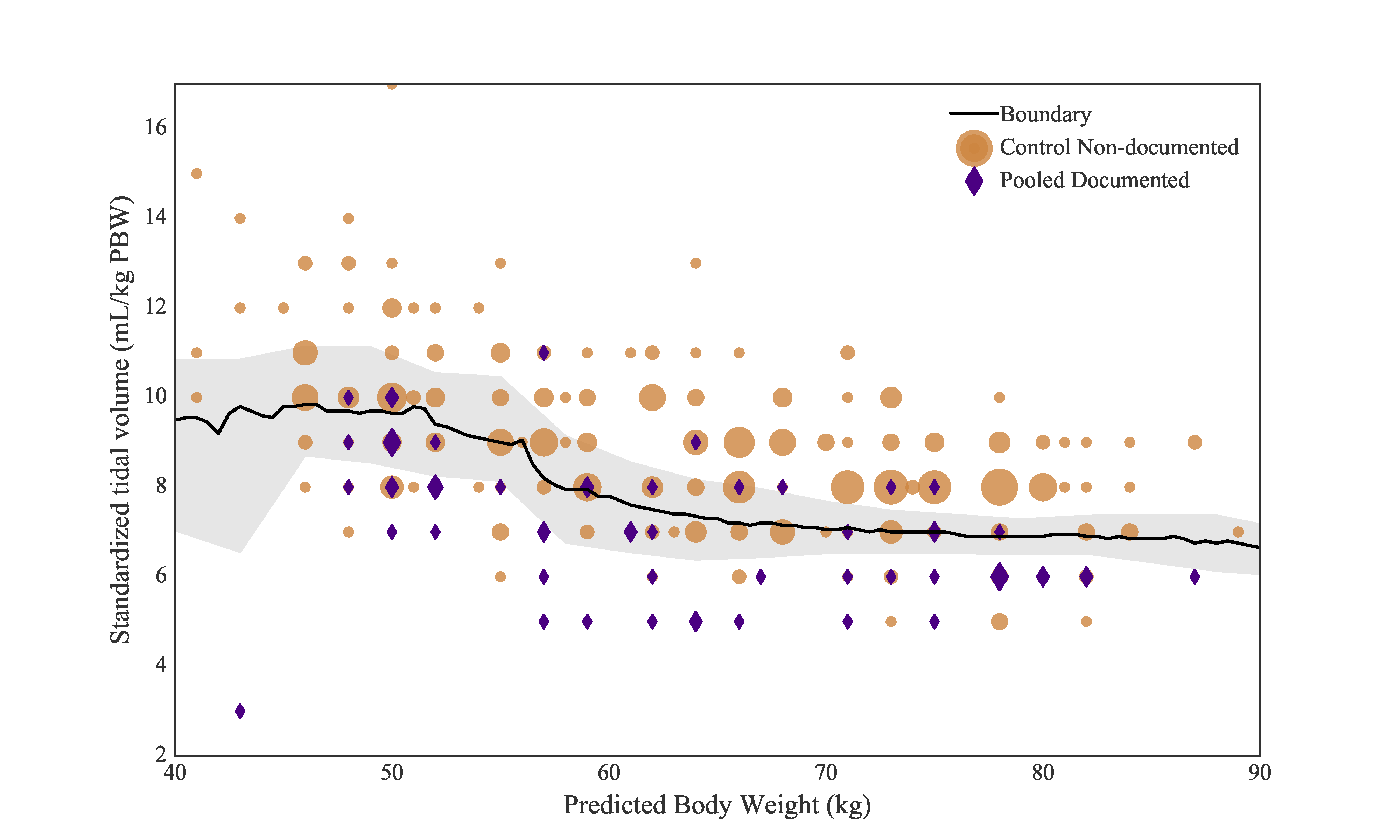

Supplement: S1 Fig — Scatter plot shows pooled documented patients (purple diamonds) and control non-documented patients (tan circles). Size of marker represents number of data points. Solid line shows boundary separating region with unequal probability of belonging to documented (below line) and non-documented control (above line) with 95% confidence bands from bootstrapped data (shaded region). (TIF) [file pone.0222826.s006.tif]

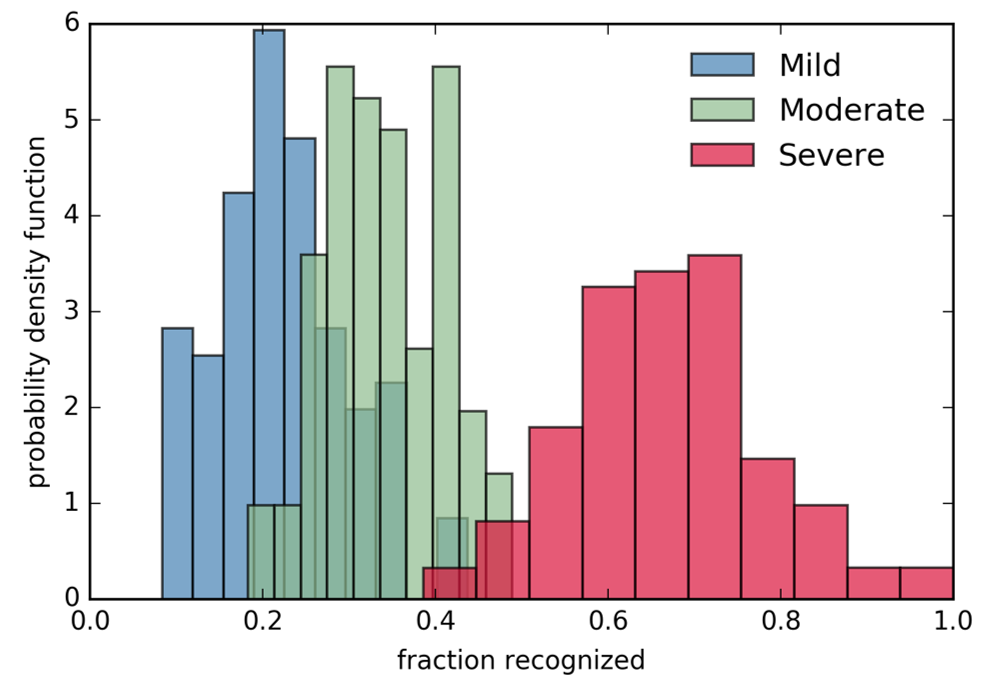

Supplement: S2 Fig — (TIF) [file pone.0222826.s007.tif]
